# Localization with Graph Diffusion Property

**DOI:** 10.3390/s17071636

**Published:** 2017-07-15

**Authors:** Pengpeng Chen, Yuqing Yin, Shouwan Gao, Qiang Niu, Jun Gu

**Affiliations:** School of Computer Science and Technology, China University of Mining and Technology, Xuzhou 221116, China; chenp@cumt.edu.cn (P.C.); yinyuqing@cumt.edu.cn (Y.Y.); gaoshouwan@cumt.edu.cn (S.G.); gujcumt@163.com (J.G.)

**Keywords:** node localization, graph diffusion property, wireless sensor networks

## Abstract

Node localization is an essential issue in wireless sensor networks (WSNs). Many range-free localization methods have been proposed to satisfy the requirement of low-system cost. However, some range-free methods only depend on network connectivity, and others only utilize the proximity information attached in neighborhood ordering. To employ the strength of the above two aspects, this paper introduces a new metric system called Combined and Weighted Diffusion Distance (CWDD). CWDD is designed to obtain the relative distance among nodes based on both graph diffusion property and neighbor information. We implement our design by embedding CWDD into two well-known localization algorithms and evaluate it by extensive simulations. Results show that our design improves the localization performance in large scale and non-uniform sensor networks, which reduces positioning errors by as much as 26%.

## 1. Introduction

Node localization is a fundamental issue in wireless sensor networks (WSNs) that has also been studied for a long time and has received many classic and improved theories [1,2,3,4] for practical applications, e.g., border security and surveillance [4], item tracking [5,6], road monitoring [7], or environment data collection [8]. Two basic methods, namely, range-based and range-free schemes, are proposed for node localization. Although the range-based localization can provide high-accuracy ranging, range-free localization may decrease system cost. Thus, the range-free method is a better choice compared with the former one.

The range-free approach uses simple sensing to localize nodes; this approach is based on the information of wireless networks, such as connectivity and proximity information. Connectivity-based localization, e.g., DV-Hop [2], may decrease system cost at the expense of accuracy. Additionally, connectivity-based localization does not perform well in unevenly distributed networks. On the other side, some ideas collect the Received Signal Strength (RSS) to obtain the proximity information. The RSS values become uncertain and irregular because of some factors, such as radio path loss, random noise, and complex terrain. This phenomenon may also increase localization errors.

Our present work is motivated by the finding that the graph diffusion property [9] of network connectivity can be applied to calculate a new metric for relative distance among node pairs. This new concept “Graph diffusion property” was developed in the bioinformatics field for protein function prediction. In protein to protein interaction (PPI) networks, the simple shortest-path metric is not suitable to apply to the fine-grained PPI graph for similarity measurement. In view of this, Lenore J. Cowen et.al., proposed diffusion state distance (DSD), a new metric based on graph diffusion property, designed to capture finer-grained distinction in proximity for transfer of functional annotation in PPI networks. The innovation point of DSD is to apply random walk to network structure, which exactly brings out the theme “graph diffusion”. Accordingly, DSD can make full use of the important knowledge encoded in the structure of the network and can tell the difference between the adjacent of two nodes and that of two other nodes. Furthermore, few methods combine both connectivity and proximity information in WSNs to design new localization methods. Hence, the need for a novel method that compensates each other’s shortcomings arises.

In this paper we first propose a network connectivity-based diffusion distance (DD), which is a new metric to indicate the relative distance between sensor pairs. With the idea of random walk [9,10], we suspect that one node can reach the remaining neighboring nodes with the same probability. Moreover, one of the neighboring nodes will continue on repeating this section to the next neighbor node within a preset step. Subsequently, we accumulate the probabilities produced by each step among random walks and extract the dissimilarity of probabilities that serves as a relative distance. Second, this study improves the existing method and introduces combined and weighted DD (CWDD). According to RSS value, the far-and-near relation between two neighbor nodes can be determined. This relationship can be converted to numbers as the weight of connected lines. Therefore, we modify the process in random walks [11]. In terms of different weight values attached on neighbor lines, one node can reach the remaining neighbor nodes with different probabilities. When two neighbor nodes are far from each other, the probability value of reaching each other will be small. Afterward, we obtain the probabilities produced by each step among random walks. We also extract the dissimilarity of probabilities to serve as a relative distance. Finally, we embed CWDD into two classic localization algorithms, i.e., DV-Hop [2] and RPA [12], to replace hops with relative distances. Extensive simulations prove that CWDD-embedded methods perform better than hop-based methods in dense and non-uniform networks and reduce the localization errors by approximately 26%. Specifically, our main contributions in this paper are as follows.

(1) Given the graph diffusion property of network connectivity, a new metric called DD is designed to indicate the relative distance between sensor node pairs.
(2) To further improve the localization performance, a refined version named CWDD is proposed by adding nodes’ proximity information that serves as the weight on connected lines in sensor networks.
(3) Extensive simulations are conducted to prove that CWDD-embedded method outperforms original methods with high accuracy.

The remaining parts of this paper are organized as follows. Section 2 briefly surveys the related work. Section 3 explains the objective of this study. The main design is elaborated in Section 4. Section 5 embeds the main design into classic algorithms, and Section 6 discusses the results from simulations. Finally, conclusions are provided in Section 7.

## 2. Related Work

Localization in WSNs is regarded as an emerging technology for numerous systems [13]. Range-based [14,15] and range-free [16,17,18,19,20] are two main types of existing methods on localization work. Lately, other methods which combine range-based or range-free principle are also available [21].

Range-based localization requires costly ranging hardware [22] and system calibration [23] support to measure the physical distance among sensor nodes precisely and then locate unknown nodes with geometric approaches, such as TOA [24], TDOA [14], and AOA [15]. The RSS localization scheme has low hardware cost which only relies on the communication module, but it has relatively poor localization performance. In all, although most of range-based localization achieves high-localization accuracy, its hardware is expensive and limits the scope of application, which is not appropriate for large-scale WSNs.

Conversely, low-system cost is researched by using range-free localization methods. On early stage, range-free localization utilizes the advantages of anchor proximity information to locate nodes, like centroid [25]. To obtain high accuracy, the network requires high anchor density, but it is not suitable for large-scale networks because of high cost of anchors. Afterward, connectivity-based range-free methods, including DV-Hop [2], RPA [12], and MDS-MAP [26], are designed to reduce the proportion of anchors and obtain a relatively high accuracy, which reduces the system cost to some extent. Moreover, these methods construct global coordinates by creating a metric for hop-based virtual distances via neighborhood sensing, which is practical for large-scale networks.

However, these methods may suffer from hop-distance ambiguity problem. Therefore, a method based on regulated neighborhood distance (RND) is proposed to address this problem by relating the proximity of two neighbors to their neighbor partitions [13]. Supposing that node *a* and node *b* are neighbors, *A* is the neighbor set of node *a* and *B* is the neighbor set of node *b*. It defines ND as neighborhood distance, where ND(a,b)=|A−A⋂B|/|A| and ND(b,a)=|B−A⋂B|/|B|. Then, RND(a,b) is denoted as the average of two NDs. This method achieves a high correlation coefficient between RND and physical distance, and performs well in uniform and dense networks. Besides, a range-free localization method beyond connectivity is proposed in [27], which excavates further information regarding neighborhood sensing. Each node can obtain a neighbor ordering that ranges from the large values of neighborhood RSS to small ones. A signature distance between a pair of nodes can be subsequently measured by comparing the neighbor sequences of node pairs. Considering non-uniform networks, the signature distance is regulated as regulated signature distance (RSD), which is a novel metric of proximity among neighbor nodes. This design remarkably improves the localization accuracy, but increases additional time complexity responsively.

Unlike the previously presented ideas, this paper introduces the CWDD, a novel distance estimation metric based on both graph diffusion property of network connectivity and neighbor information. Given that CWDD is embedded into classic range-free localization methods, our design performs well in accuracy and can be applied for large-scale and non-uniform networks.

## 3. Motivation

We first elaborate the drawbacks of connectivity-based localization and then show the opportunity for our new method.

### 3.1. Limitations of Hop-Based Localization

Three typical connectivity-based localization approaches, namely, DV-Hop [2], MDS-MAP [12], and RPA [26], are available. The common idea of these approaches is the usage of the shortest path based on hops among node pairs. Afterward, the shortest paths are transferred into relative distances, and coordinates are estimated by the least square method. Considering DV-Hop as an example, distance vector routing and theory of GPS localization are used. The algorithm is divided into the following steps:

(1) In all nodes, the shortest hops reaching each anchor are calculated. $Hops(u_{i},v_{j})$ represents the least number of hops between an unknown node $u_{i}$ and an anchor $v_{j}$.
(2) Utilizing the anchors’ coordinates, the algorithm can estimate the average physical distances for one hop using the following Equation (1):
  (1) $$HopSize=\frac{\sum_{i\neq j}^{}\sqrt{(x_{i}-x_{j}{{)}^{2}+(y_{i}-y_{j})}^{2}}}{\sum_{i\neq j}^{}Hops\left(v_{i},v_{j}\right)}$$
  where $v_{i}\left(x_{i},y_{i}\right)$ and $v_{j}\left(x_{j},y_{j}\right)$ are anchors, and $Hops(v_{i},v_{j})$ is the number of the least hops between $v_{i}$ and $v_{j}$. For each unknown node $u_{i}$, the distances of reaching each anchor $v_{j}$ can be estimated using the formula $HopSize\times Hops(u_{i},v_{j})$.
(3) Along with anchors’ coordinates and distances to all anchors, an unknown node will finally determine its coordinate using maximum likelihood estimation method.

The drawback of this algorithm is that hop size only stands for a mean distance for one hop, which may get fair precision in uniform networks because of similar distances among hops. However, this hop is not appropriate especially for non-uniform networks. Hop-based algorithm will cause large localization errors, because one hop may corresponds several distances.

### 3.2. Opportunity for Our New Method

**Figure 1:**
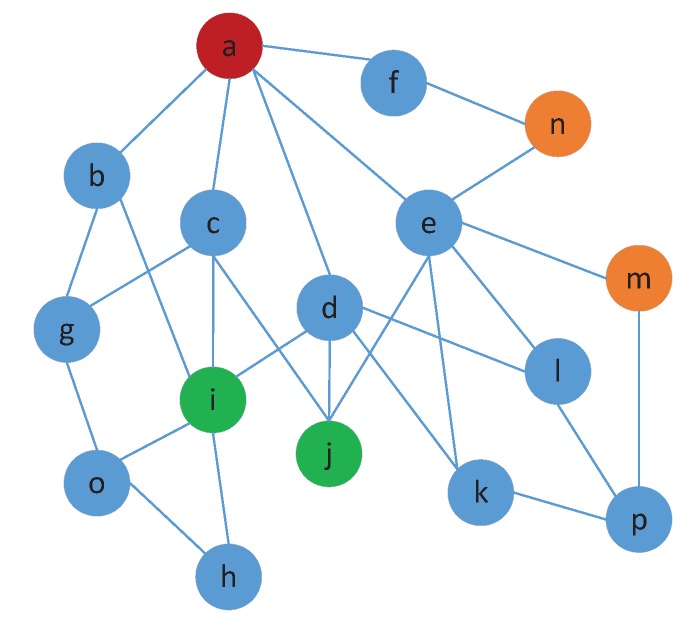
Different number of common neighbors showing different distances.

Considering the limitation investigated in the last section, we bypass the hop-distance model by analyzing some other useful information, such as graph diffusion property from network connectivity. As illustrated in Figure 1, two remarkable observations are obtained.

**Remark** **1.** In wireless sensor networks, two nodes with many neighbors in common are closer than nodes with few neighbors in common.

**Remark** **2.** Such two nodes with many common low-degree neighbors are closer than nodes with high-degree common neighbors.

**Figure 2:**
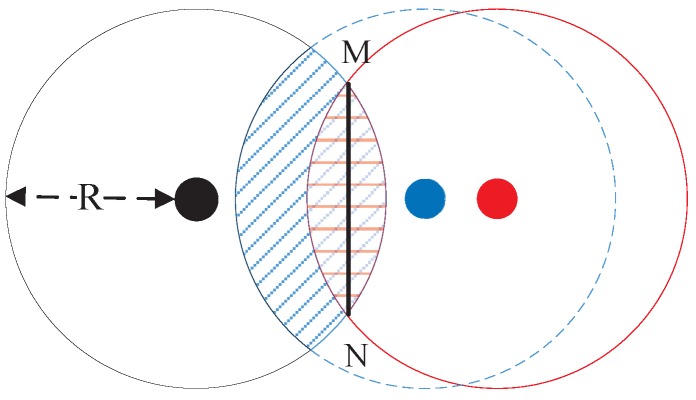
The diagrammatic sketch of Remarks 1 & 2.

As shown in Figure 2, there are three nodes with radio range *R*. The common neighbors can only exist in the overlapping region of two large circles. Therefore, the overlapping region is large with the presence of many common neighbors in a node pair, and the distance within a node pair is close when the two centers of circles are close to each other, which explains why Remark 1 makes sense.

Remark 2 leads us to think about the following question. Under the condition of same quantity of common neighbor nodes between two nodes, which types of common neighbor nodes the two nodes have more, low-degree nodes or high-degree nodes, can shorten the distance between these two nodes? Assuming that there are fixed number of common neighbors in the overlapping region. One common neighbor can treat all other common neighbors in the overlapping region as its neighbor, when the intersecting chord of two circles like segment MN in Figure 2 (the longest connection range of two common neighbors) is shorter than radio range *R*. On the other hand, when the intersecting chord of two circles is longer than radio range *R*, there may exit that one common neighbor cannot connect with some other nodes in the overlapping region, which may relatively reduce the degree of this common neighbor compared with the former case to some extent. To sum up, low-degree common neighbors appear in the larger overlapping region than relatively high-degree common neighbors do, and according to the explanations of Remark 1, larger overlapping region draw two nodes more closely.

A connectivity graph of a network is observed by comparing the nodes in Figure 1. As concluded from Remark 1, the distance between nodes *a* and *n* is smaller than that of nodes *a* and *m* because nodes *a* and *n* exhibit two common neighbors, namely, nodes *f* and *e*. Nodes *a* and *m* share node *e* only. Furthermore, the degrees of nodes *c*, *d*, and *e* are 4, 5, and 6, respectively. According to Remark 2, the distance between nodes *a* and *i* is smaller than that of nodes *a* and *j* because nodes *a* and *i* display relatively low-degree common neighbors. By contrast, nodes *a* and *j* show relatively high-degree common neighbors.

According to the property of network connectivity mentioned above, we first propose a DD, which is a new metric to indicate the relative distance between node pairs. With the limitation of hop-based localization, we regard proximity information as a weight attached on connective line for one hop. Therefore, we prove the method and introduce CWDD by combining the properties of network connectivity and proximity information.

## 4. System Design

### 4.1. Definition of a New Distance Metric

On a 2D plane, we consider locating a network of wireless nodes with connectivity information as the undirected graph G(V,E), where $V=\left\{v_{1},v_{2},...,v_{n}\right\}$ is the vertex set, and *E* is the adjacency matrix of this graph.

Given the adjacency matrix of graph *G*, a probability matrix for one-hop neighbors *P* can be obtained with the following steps, where the (i,j)th entry is given by Equation (2).

(1) The value of each row in adjacency matrix *E* is summed as the degree of each node.
(2) Each row of *E* is divided with the corresponding degree.

(2) $$P\left(i,j\right)=\left\{\begin{array}{cc} \frac{1}{\sum_{l=1}^{n}E\left(v_{i},v_{l}\right)} & \;\mathrm{if}\;E(v_{i},v_{j})=1\\ \;0 & \;\mathrm{if}\;E(v_{i},v_{j})=0 \end{array}\right.$$

Considering the concept of one-hop probability matrix *P*, any node $v_{i}$ will reach its next one-hop neighbors with the same probability, which is determined by the degree of node $v_{i}$. Subsequently, $p^{\left(l\right)}\left(v_{i},v_{j}\right)$ is defined as the probability from node $v_{i}$ proceeding *l* steps toward node $v_{j}$ in a random walk. Due to each step of random walk is an independent event, the probability of each path from node $v_{i}$ proceeding *l* steps toward node $v_{j}$ can be expressed as the product of all one-hop probabilities along this path. Furthermore, $p^{\left(l\right)}\left(v_{i},v_{j}\right)$ comes out by adding all probabilities of each path. $Ap^{\left(m\right)}\left(v_{i},v_{j}\right)$ is denoted as the accumulated probability produced from one step to *m* steps in a random walk starting at node $v_{i}$ and finishing at node $v_{j}$, where

(3) $$Ap^{\left(m\right)}\left(v_{i},v_{j}\right)=\sum_{l=1}^{m}p^{\left(l\right)}\left(v_{i},v_{j}\right)$$

We further define a *n*-dimensional vector $Apv^{\left(m\right)}\left(v_{i}\right),\;\forall vi\in V$, where

(4) $$\begin{array}{ccc} Apv^{\left(m\right)}\left(v_{i}\right)= & \left\{Ap^{\left(m\right)}\left(v_{i},v_{1}\right),Ap^{\left(m\right)}\left(v_{i},v_{2}\right),\right. & \left....,Ap^{\left(m\right)}\left(v_{i},v_{n-1}\right),Ap^{\left(m\right)}\left(v_{i},v_{n}\right)\right\} \end{array}$$

A DD between two vertices $v_{i}$ and $v_{j}$, $\forall v_{i},v_{j}\in V$ is also denoted as follows:

(5) $$DD^{\left(m\right)}\left(v_{i},v_{j}\right)={∥Apv^{\left(m\right)}\left(v_{i}\right)-Apv^{\left(m\right)}\left(v_{j}\right)∥}_{1}$$

where ${∥...∥}_{1}$ means the $L_{1}$ norm of two vectors’ difference.

**Figure 3:**
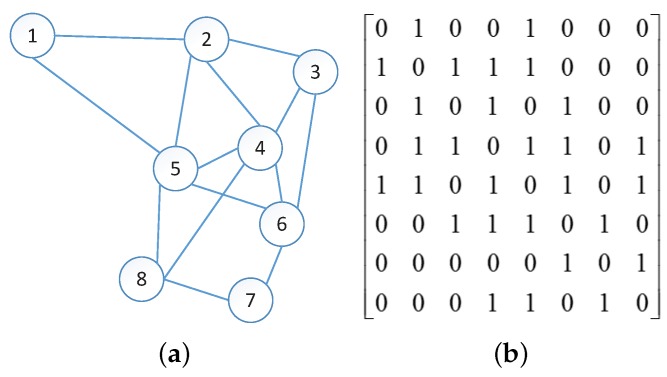
Diffusion Distance (DD): (**a**) Topological graph $G_{0}$, (**b**) Adjacency matrix *E*.

Figure 3 shows a simple example, including a topological graph $G_{0}$ of a wireless network on the left and the corresponding adjacency matrix *E* on the right. We use the connectivity information illustrated in Figure 3 to calculate the value of $DD^{\left(2\right)}\left(v_{1},v_{4}\right)$ and $DD^{\left(2\right)}\left(v_{1},v_{6}\right)$ with the assumption m=2.

First, the result of one-hop probability matrix *P* is the following matrix.

$$\left[\begin{array}{cccccccc} 0 & 1/2 & 0 & 0 & 1/2 & 0 & 0 & 0\\ 1/4 & 0 & 1/4 & 1/4 & 1/4 & 0 & 0 & 0\\ 0 & 1/3 & 0 & 1/3 & 0 & 1/3 & 0 & 0\\ 0 & 1/5 & 1/5 & 0 & 1/5 & 1/5 & 0 & 1/5\\ 1/5 & 1/5 & 0 & 1/5 & 0 & 1/5 & 0 & 1/5\\ 0 & 0 & 1/4 & 1/4 & 1/4 & 0 & 1/4 & 0\\ 0 & 0 & 0 & 0 & 0 & 1/2 & 0 & 1/2\\ 0 & 0 & 0 & 1/3 & 1/3 & 0 & 1/3 & 0 \end{array}\right]$$

Second, the accumulated probability can be calculated as follows:

$$\begin{array}{cc} Ap^{\left(2\right)}\left(v_{1},v_{4}\right) & =p^{\left(1\right)}\left(v_{1},v_{4}\right)+p^{\left(2\right)}\left(v_{1},v_{4}\right)\\ & =0+9/40\approx 0.225, \end{array}$$

where 9/40=1/2×1/4+1/2×1/5, means that two paths exist from $v_{1}$ to $v_{4}$ through $v_{2}$ and $v_{5}$ during the two steps. Similarly,

$$Ap^{\left(2\right)}\left(v_{1},v_{6}\right)=p^{\left(1\right)}\left(v_{1},v_{6}\right)+p^{\left(2\right)}\left(v_{1},v_{6}\right)=0+0.1=0.1.$$

Third, we obtain three *n*-dimensional vectors, namely, $Apv^{\left(2\right)}\left(v_{1}\right)$, $Apv^{\left(2\right)}\left(v_{4}\right)$, and $Apv^{\left(2\right)}\left(v_{6}\right)$.

$$\begin{array}{c} \;Apv^{\left(2\right)}\left(v_{1}\right)\\ =\left\{Ap^{\left(2\right)}\left(v_{1},v_{1}\right),Ap^{\left(2\right)}\left(v_{1},v_{2}\right),...,Ap^{\left(2\right)}\left(v_{1},v_{8}\right)\right\}\\ =\left\{0.225,0.6,0.125,0.225,0.625,0.1,0,0.1\right\} \end{array}$$

$$\begin{array}{c} \;Apv^{\left(2\right)}\left(v_{4}\right)\\ =\left\{Ap^{\left(2\right)}\left(v_{4},v_{1}\right),Ap^{\left(2\right)}\left(v_{4},v_{2}\right),...,Ap^{\left(2\right)}\left(v_{4},v_{8}\right)\right\}\\ =\left\{0.09,0.31,0.3,0.27,0.37,0.31,0.12,0.24\right\} \end{array}$$

$$\begin{array}{c} \;Apv^{\left(2\right)}\left(v_{6}\right)\\ =\left\{Ap^{\left(2\right)}\left(v_{6},v_{1}\right),Ap^{\left(2\right)}\left(v_{6},v_{2}\right),...,Ap^{\left(2\right)}\left(v_{6},v_{8}\right)\right\}\\ =\left\{0.05,0.18,0.3,0.38,0.3,0.31,0.25,0.225\right\} \end{array}$$

Finally, $DD^{\left(2\right)}\left(v_{1},v_{4}\right)$ and $DD^{\left(2\right)}\left(v_{1},v_{6}\right)$ are counted as follows.

$$\begin{array}{cc} DD^{\left(2\right)}\left(v_{1},v_{4}\right) & ={∥Apv^{\left(2\right)}\left(v_{1}\right)-Apv^{\left(2\right)}\left(v_{4}\right)∥}_{1}\\ & =|0.225-0.09|+|0.6-0.31|\\ & \;+|0.125-0.3|+|0.225-0.27|\\ & \;+|0.625-0.37|+|0.1-0.31|\\ & \;+|0-0.12|+|0.1-0.24|\\ & =1.36 \end{array}$$

$$\begin{array}{cc} DD^{\left(2\right)}\left(v_{1},v_{6}\right) & ={∥Apv^{\left(2\right)}\left(v_{1}\right)-Apv^{\left(2\right)}\left(v_{6}\right)∥}_{1}\\ & =|0.225-0.05|+|0.6-0.18|\\ & \;+|0.125-0.3|+|0.225-0.38|\\ & \;+|0.625-0.3|+|0.1-0.31|\\ & \;+|0-0.25|+|0.1-0.225|\\ & =1.88 \end{array}$$

$L_{1}$ norm counts the absolute value of two vectors’ difference. Thus, the definition of this new distance metric guarantees $DD^{\left(m\right)}\left(v_{i},v_{j}\right)\equiv DD^{\left(m\right)}\left(v_{j},v_{i}\right)$, assuming that the steps of random walks *m* are fixed. Moreover, we can conclude that $DD^{\left(m\right)}\left(v_{i},v_{i}\right)\equiv 0$ from Equation (5).

**Algorithm 1:** Diffusion Distance.  **Input**: $\mathrm{adj\_m},i,j=\left\{1,2,...,N\right\}$  **Output**: DD_m1:degree(i)=sum(adj_m(i,:));2:P(i,:)=adj_m(i,:)/degree(i);3:${\mathrm{Ap\_m}}^{\left(m\right)}=P+P^{2}+...+P^{m};$4:$\mathrm{DD\_m}\left(i,j\right)={∥{\mathrm{Ap\_m}}^{\left(m\right)}\left(i,:\right)-{\mathrm{Ap\_m}}^{\left(m\right)}\left(j,:\right)∥}_{1}.$



Algorithm 1 shows the entire process for computing a DD matrix, where the (i,j)th entry represents the relative distance between nodes $v_{i}$ and $v_{j}$. Initially, the adjacency matrix of graph $G_{0}$ shortened as adj_m is needed as an input. For $i=\left\{1,2,3,...,N\right\},degree\left(i\right)=sum\left(adj\_m\left(i,:\right)\right)$ counts the degree of each node with the complexity O(N), where *N* is the number of all nodes. Subsequently, for $i=\left\{1,2,...,N\right\}$, one-hop probability matrix *P* consists of P(i,:)=adj_m(i,:)/degree(i) with the cost of O(N). The accumulated probability matrix $Ap\_m^{\left(m\right)}$ can be calculated by formula $Ap\_m^{\left(m\right)}=P+P^{2}+...+P^{m}$, where the ith row or column is coincident with the *n*-dimensional vector $Apv^{\left(m\right)}\left(v_{i}\right)$. Finally, the DD matrix DD_m is composed of N×N entries. The (i,j)th entry is the result of $L_{1}$ norm of the difference between the ith and jth rows of $Ap\_m^{\left(m\right)}$ based on Equation (5) with complexity $O(N^{3})$, where the values on diagonal line of DD_m are all zeros.

### 4.2. Insight into the DD

This section discusses how DD can serve as relative distance and what DD is going to be when *m* tends to infinity.

Recalling Remark 1 from Section 3, two nodes with many neighbors in common are closer than nodes with few neighbors in common. On the basis of the example above, node pair $\left(v_{1},v_{4}\right)$ presents two common neighbors, which is more than one common neighbor between node pair $\left(v_{1},v_{6}\right)$. Thus, $v_{4}$ is closer to $v_{1}$ than $v_{6}$. Furthermore, the DDs between $\left(v_{1},v_{4}\right)$ and $\left(v_{1},v_{6}\right)$ node pairs, which are counted as following formulas, are 1.36 and 1.88, respectively. These two numbers reflecting far and near relation among node pairs are also coincident with this remark.

$$\begin{array}{cc} DD^{\left(2\right)}\left(v_{1},v_{4}\right) & ={∥Apv^{\left(2\right)}\left(v_{1}\right)-Apv^{\left(2\right)}\left(v_{4}\right)∥}_{1}\\ & =|0.225-0.09|+\underline{\left|0.6-0.31\right|}\\ & \;+|0.125-0.3|+|0.225-0.27|\\ & \;+\left|0.625-0.37\right|+|0.1-0.31|\\ & \;+|0-0.12|+|0.1-0.24|\\ & =1.36 \end{array}$$

$$\begin{array}{cc} DD^{\left(2\right)}\left(v_{1},v_{6}\right) & ={∥Apv^{\left(2\right)}\left(v_{1}\right)-Apv^{\left(2\right)}\left(v_{6}\right)∥}_{1}\\ & =|0.225-0.05|+\underline{\left|0.6-0.18\right|}\\ & \;+|0.125-0.3|+|0.225-0.38|\\ & \;+\left|0.625-0.3\right|+|0.1-0.31|\\ & \;+|0-0.25|+|0.1-0.225|\\ & =1.88 \end{array}$$

The underlined items (the second ones) mean the absolute values of formulas:

$$Ap^{\left(2\right)}\left(v_{1},v_{2}\right)-Ap^{\left(2\right)}\left(v_{4},v_{2}\right),$$

$$Ap^{\left(2\right)}\left(v_{1},v_{5}\right)-Ap^{\left(2\right)}\left(v_{4},v_{5}\right);$$

The bold items (the fifth ones) mean the absolute values of formulas:

$$Ap^{\left(2\right)}\left(v_{1},v_{2}\right)-Ap^{\left(2\right)}\left(v_{6},v_{2}\right),$$

$$Ap^{\left(2\right)}\left(v_{1},v_{5}\right)-Ap^{\left(2\right)}\left(v_{6},v_{5}\right);$$

where $v_{2}$, $v_{5}$ are the common neighbors of node pair $\left(v_{1},v_{4}\right)$ and $v_{5}$ is the common neighbor of node pair $\left(v_{1},v_{6}\right)$. When the same common neighbor $v_{5}$ is shared, the values of two bold items (the fifth ones) are almost same. However, two underlined items (the second ones) show different values according to the existence of a common neighbor $v_{2}$. The difference value of sharing $v_{2}$ in common is smaller than that of not having $v_{2}$ in common. Consequently, difference calculation of the accumulated probability vectors can shorten the DD of node pairs with many common neighbors, thereby the conclusion is shown as Equation (6) based on Remark 1:

(6) $$DD^{\left(m\right)}\left(v_{i},v_{j}\right)\propto PD\left(v_{i},v_{j}\right)$$

where PD represents the physical distance. This new distance metric, DD, can be a relative distance because of this approximately proportional relation. Further information on how to convert relative distance to physical distance is introduced in Section 5.

Furthermore, we are going to discuss the DD value under the condition that random walk step *m* tends to infinity. There is a remarkable characteristic of the random walk model, that is, the result of each iteration is only related to the previous one, and it has nothing to do with the earlier results. This process is also known as Markov process or Markov chain. Thus, there exists a positive integer k(k<m), such that matrix $P^{k}=P^{k+1}=...=P^{m}$ when *m* becomes infinity in a random walk. These same parameters may be removed when making difference calculation of accumulated probability vectors. Consequently, the DD value may converge to the extreme value as *m* goes to infinity. Let $M=\lim_{m\to \infty }P^{m}$ is the constant matrix in which each row is a copy of the unique steady state distribution [28]. According to Algorithm 1, we can derive the stable value of DD between node $v_{i}$ and node $v_{j}$ as follows:

$$\begin{array}{c} \lim_{m\to \infty }DD^{\left(m\right)}\left(v_{i},v_{j}\right)\\ ={∥\lim_{m\to \infty }Apv^{\left(m\right)}\left(v_{i}\right)-Apv^{\left(m\right)}\left(v_{j}\right)∥}_{1} \end{array}$$

(7) $$\begin{array}{c} ={∥\lim_{m\to \infty }\left(b_{i}^{T}-b_{j}^{T}\right)\left(P+P^{2}+\cdots +P^{m}\right)∥}_{1} \end{array}$$

(8) $$\begin{array}{c} ={∥\lim_{m\to \infty }\left(b_{i}^{T}-b_{j}^{T}\right)\frac{P(1-P^{m})}{1-P}∥}_{1} \end{array}$$

(9) $$\begin{array}{c} =\left(b_{i}^{T}-b_{j}^{T}\right)P\left(1-M\right){\left(1-P\right)}^{-1} \end{array}$$

where $b_{i}$ is the ith basis vector, i.e., the row vector of all zeros except for a 1 in the ith position [28]. For deeper interpretation, Equation (7) holds by step 3 in Algorithm 1, Equation (8) utilizes the summation formula of geometric series, and Equation (9) forms the final invariable value of DD.

### 4.3. CWDD Design

In the above analysis, we only make use of network connectivity for the random walk. However, the neighborhood proximity information behind RSS values can also affect the distance between two nodes. In addition, it is not accurate to consider that one node can reach to each of its next-hop nodes with same probabilities. In view of these reasons, we propose a refined version of DD, that is, combined and weighted diffusion distance (CWDD). Just as the name says, CWDD combines network connectivity and neighborhood proximity these two factors by taking proximity information as a weight value attached on connective lines. The weight values can distinguish the probabilities from one node to its neighbor nodes, and then CWDD can tell the tiny differences among several distances, which can improve the positioning accuracy to some extent. We use the modified RSD [27] as the weight. Recalling the introduction from Section 2, a signature distance can be first obtained from RSS values and then regulated to RSD with Equation (10):

(10) $$RSD\left(v_{i},v_{j}\right)=SD\left(S_{i},S_{j}\right)\frac{\sqrt{K}}{K(K-1)/2}$$

where $S_{i}$ is the neighbor ordering of node $v_{i}$, and $K=|S_{i}\cup S_{j}|$ is the total number of nodes in the neighborhood of nodes $v_{i}$ and $v_{j}$.

Subsequently, we reconstruct the undirected graph $G(V_{conb},E_{conb},W)$, where $V_{conb}=V$ is the vertex set, and $E_{conb}=E$ is the adjacency matrix. *W* is the weight matrix, where the (i,j)th entry is described as follows:

(11) $$w_{i,j}=1-SD\left(S_{i},S_{j}\right)\frac{2}{K(K-1)}$$

Equation (11) is a modified RSD, which satisfies the condition that the weight value is inversely proportional to physical distance. This process is in preparation for one-hop probability matrix $P_{conb}$, where the (i,j)th entry is given by the following:

(12) $$P_{comb}\left(i,j\right)=\left\{\begin{array}{cc} \frac{w_{i,j}}{\sum_{l=1}^{n}w_{i,l}} & \;\mathrm{if}\;E(v_{i},v_{j})=1\\ \;\;\;0\; & \;\mathrm{if}\;E(v_{i},v_{j})=0 \end{array}\right.$$

Equation (12) implies the small distance between $v_{i}$ and its one-hop neighbor $v_{j}$ and the high probability proceeding from $v_{i}$ to $v_{j}$ in a random walk. Accordingly, ${p_{conb}}^{\left(m\right)}\left(v_{i},v_{j}\right)$ is constructed as the probability from node $v_{i}$ proceeding *m* steps toward node $v_{j}$, and $A{p_{conb}}^{\left(m\right)}\left(v_{i},v_{j}\right)$ is the accumulated probability. Therefore, the *n*-dimensional vector $Ap{v_{conb}}^{\left(m\right)}\left(v_{i}\right)$, $\forall v_{i}\in V$ can be obtained, and the definition of CWDD is shown as Equation (13).

(13) $$\begin{array}{ccc} & \;CWDD^{\left(m\right)}\left(v_{i},v_{j}\right) & ={∥Ap{v_{conb}}^{\left(m\right)}\left(v_{i}\right)-Ap{v_{conb}}^{\left(m\right)}\left(v_{j}\right)∥}_{1} \end{array}$$

Thus, we introduce the new metric for relative distance and its combined version applying for node localization in WSNs.

## 5. Embedding Design

The design of new metrics for relative distance can be embedded into two classical connectivity-based schemes for localizing the coordinates of unknown nodes. These nodes are compared with real coordinates to evaluate the system accuracy, and the two schemes are as follows:

- DV-Hop [2] by D. Niculescu and B. Nath.
- RPA [12] by C. Savarese, J.M. Rabary et al.

DV-Hop is described in Section 3, and a brief description of RPA algorithm is described in this section. RPA is separated into two periods, namely, start-up and refinement. For the first phase, Hop-TERRAIN, an in-house algorithm similar to DV-Hop, is used for allowing all nodes to arrive at initial position estimates by cooperatively spreading awareness of the anchor nodes’ positions throughout the network. The second phase proposes an iterative refinement algorithm for position adjustment based on the results of first step because of the low accuracy of initial estimates. In addition, at iteration *k*, refinement is concerned only with nodes existing within a one-hop neighborhood, and it is recomputed based on the estimated positions from iteration k−1. RPA aims to increase the system accuracy. However, the high number of iterations will result in further time complexity in the system costs.

Considering the challenge of hop-based design, the value of new metric for relative distance can be used to replace the shortest-path hop distance. For DV-Hop, the expected physical distance for one unit relative distance (DD or CWDD) can be modified based on Equation (1) in Section 3, which is given by Equation (14).

(14) $$RD_{unit}Size=\frac{\sum_{i\neq j}\sqrt{{\left(x_{i}-x_{j}\right)}^{2}+{\left(y_{i}-y_{j}\right)}^{2}}}{\sum_{i\neq j}RD(v_{i},v_{j})}$$

For RPA, Equation (14) is also suitable for the revision of the first phase. Moreover, the refinement step also benefits from embedding DD or CWDD for adjusting iteration position.

## 6. Simulation Evaluation

In this section, we report different simulation results about several aspects, such as the correlation between relative distance and physical distance, discussion of the effects with different random-walk steps, and localization performance under different settings with and without CWDD embedding.

### 6.1. Default Simulation Configurations

In the simulation, we create a WSN as a square map without holes, which means that radio signal can reach anywhere in its area of interest. Further complicated maps can be used with works [29,30,31]. Unless otherwise specified, default simulation configurations are listed in Table 1 for the entire section.

Table 1 shows that noise model is a widely used logarithmic attenuation model [32,33] for RSS sensing, where the sensing result between two nodes is given by Equation (15):

(15) $$P_{i,j}\left(t\right)=P_{t}-P_{l}\left(d_{0}\right)-10\beta \log\left(\frac{PD(v_{i},v_{j})}{d_{0}}\right)+X_{i}\left(t\right)$$

where $P_{i,j}\left(t\right)$ stands for the sensing result starting from node $v_{i}$ to node $v_{j}$ at time instance *t*, $P_{t}$ is the transmit power at a short reference distance $d_{0}$, $P_{l}\left(d_{0}\right)$ is the pass loss for the reference distance $d_{0}$, β means the pass loss exponent or fading factor and $PD(v_{i},v_{j})$ is the physical distance between sender node $v_{i}$ and receiver node $v_{j}$, and $X_{i}\left(t\right)$ means that for node $v_{i}$ it follows $X_{i}\left(t\right)∼N\left(0,\sigma_{X}^{2}\right)$ at time *t*.

Furthermore, we define system localization error LE as follows:

(16) $$LE=\frac{\sum_{i=1}^{n}\sqrt{{\left(x_{ie}-x_{ir}\right)}^{2}+{\left(y_{ie}-y_{ir}\right)}^{2}}}{N_{non\_anc}\times R}$$

where *n* is the number of nodes, $\left(x_{ie},y_{ie}\right)$ is the estimated coordinate of node $v_{i}$, and $\left(x_{ir},y_{ir}\right)$ is the real coordinate of node $v_{i}$, $N_{non\_anc}$ means the number of non-anchor nodes, and *R* represents the radio range. For high confidence, all following statistics of localization errors are averaged over 50 runs.

### 6.2. Distance Correlations

**Figure 4:**
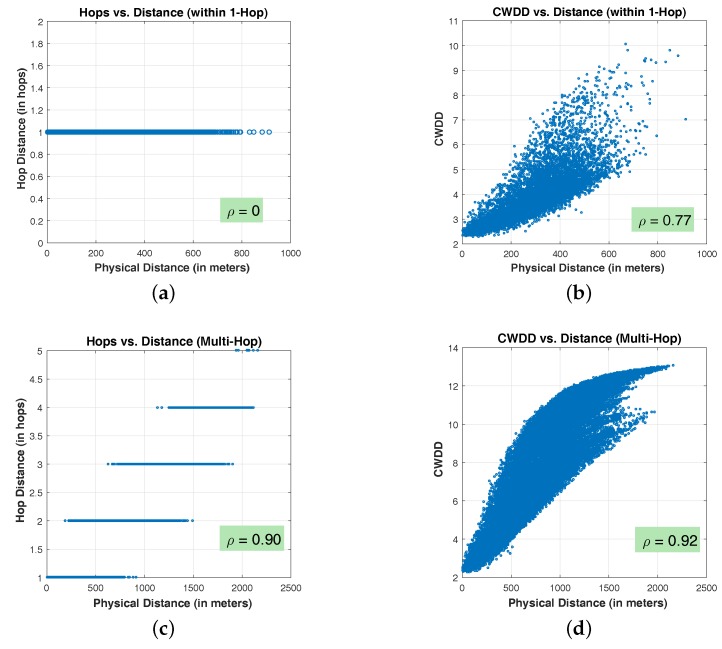
Distance correlation comparison: Hop vs. DD: (**a**) hops vs. distance (within 1-hop), (**b**) CWDD vs. distance (within 1-hop), (**c**) hops vs. distance (multi-hop), (**d**) CWDD vs. distance (multi-hop).

Figure 4 shows the correlations between hop-based distance, DD, and the physical distance. Figure 4a,b plot the node pairs within one-hop connectivity in networks. Figure 4c,d plot all the node pairs, including one-hop and multi-hop node pairs and their shortest-path hops and DD distance against physical distance. In these four figures, X axis represents the physical distance among node pairs, and Y axis represents the hop distance and DD.

Figure 4a indicates that node pairs within one-hop connectivity exhibit an identical hop distance “1”, regardless of the corresponding physical distances. Consequently, correlation coefficient ρ is 0. By contrast, as illustrated in Figure 4b, a roughly linear correlation exists between DD value and physical distance, where the correlation coefficient is ρ=0.77. The new metric for relative distance provides a sub-hop resolution that is not available in hop-based methods. Comparing Figure 4c,d, the correlation coefficients are close, where ρ=0.90 is for hop-based distance, and ρ=0.92 is for DD. The latter figure shows that DD provides improved resolution. Figure 4c indicates that only discrete integer hop distances exist among node pairs, and Figure 4d shows that physical distances can be mapped to continuous values.

In addition, Figure 4b,d confirm the remark analyzed in Section 4 that DD is approximately proportionally related to physical distance.

### 6.3. DD vs. CWDD

Table 2 is obtained from the simulation with default configurations and compares the localization performances with DD and CWDD serving as a relative distance. Node deviation is the Euclidean distance between expected nodes’ coordinate and real nodes’ coordinate. CWDD proves localization precision, and it is a better metric of relative distance than DD.

### 6.4. Localization Performance

This subsection provides the localization results by comparing the images and statistics between two types of localization methods (without and with CWDD embedding).

**Figure 5:**
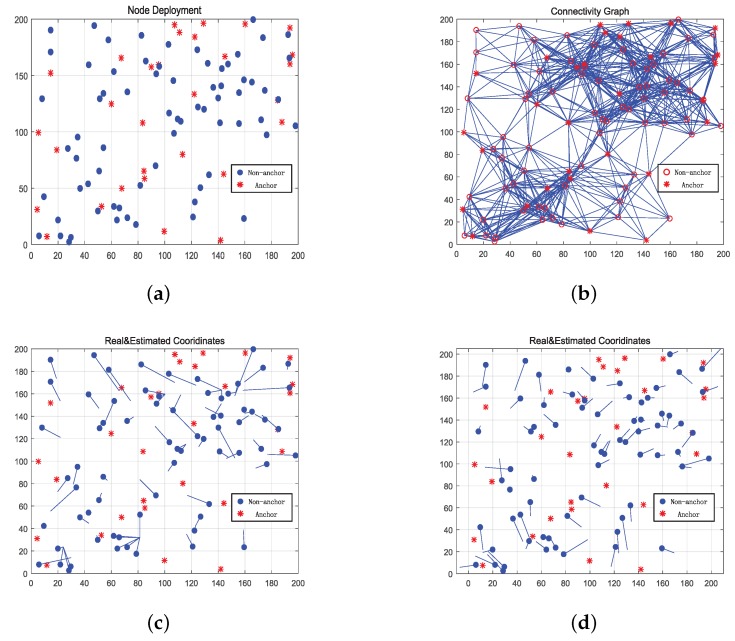
Localization results of DV-Hop and DV-CWDD: (**a**) node deployment, (**b**) connectivity graph, (**c**) DV-Hop, (**d**) DV-CWDD.

For improved vision effect, we reduce the scale of the network in this simulation as a 200 m × 200 m map. As shown in Figure 5a, 100 nodes (30 red asterisks for the anchors, and 70 blue dots for the nodes to be located) are randomly deployed in this non-uniform network. With the radio range R=50, Figure 5b presents the connectivity of the system. Connected lines are clustered together in the upper right quarter and lower left quarter parts of Figure 5b. In addition, nodes in Figure 5a are considerably clustered around these two areas.

Figure 5c,d display the localization results from DV-Hop and DV-CWDD, respectively. In both figures, the red asterisks are anchors’ positions, blue dots are the real positions of unknown nodes, and the blue line segments are directing to the estimated positions. Long segment results in large prediction error. According to Figure 5c,d , it can be shown that:

(1) DV-CWDD performs better than DV-Hop as most line segments in Figure 5d are shorter than those in Figure 5c.
(2) DV-Hop may cause clustered mapping. Therefore, some nodes are mapped to the same estimated position. The different distances between node pairs are mapped to the identical hop distance, thereby causing an ambiguity problem. However, this type of issues cannot be observed evidently in DV-CWDD.
(3) In the upper right quarter of Figure 5d, CWDD obtains higher localization accuracy than that in other areas.

**Figure 6:**
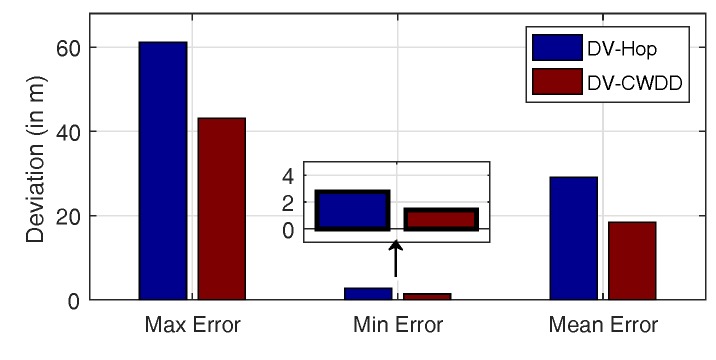
Comparison among maximum, minimum, and mean deviations.

Figure 6 shows the maximum, minimum, and mean deviations from the results of simulations for DV-Hop and DV-CWDD. All statistics in this bar chart are averaged by over 100 runs for high reliability. Three red bars are a third less than blue bars. This finding is a good confirmation that CWDD offers an effective improvement in localization accuracy.

Localization results prove that CWDD method provides an effective resolution for localization precision and ambiguity problem. Moreover, this method performs well in non-uniform networks, especially in dense areas.

### 6.5. Effectiveness of CWDD

This subsection evaluates the new distance metric by comparing localization errors among hop-based methods (DV-Hop and RPA-Hop), CWDD-embedded methods (DV-CWDD and RPA-CWDD) under different configurations.

#### 6.5.1. Effect of Random-Walk Steps

**Figure 7:**
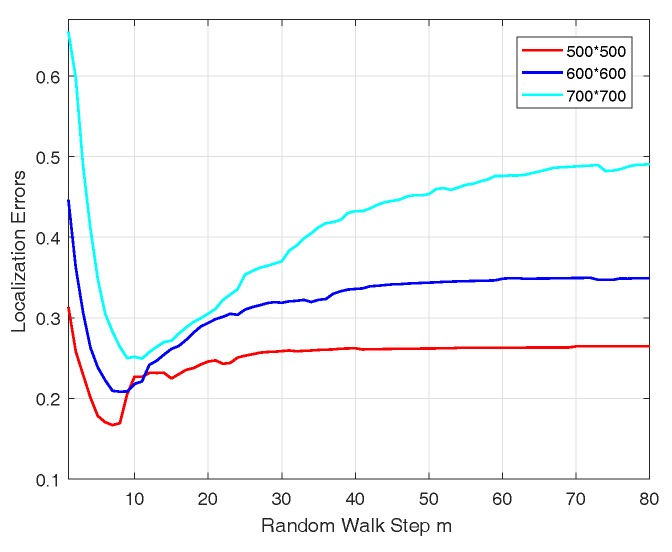
Localization errors with variable *m*.

DD is not constant because of random walk step *m*. When *m* is fixed, CWDD is considered a relative distance proportional to real distance. Nevertheless, localization accuracy may change with variable *m*. Figure 7 shows the localization errors of DV-CWDD varying with *m* under three system scales (500 × 500, 600 × 600, and 700 × 700) with 130 m radio range.

As illustrated in Figure 7, each line exhibits a valley near the front part, and the corresponding *m* is marked as $m_{best}$. Localization accuracy achieves the most suitable result at $m_{best}$. Prior to $m_{best}$, the lines decline when $m_{best}$ lines present the upward trend and stabilize gradually. Numerous simulations concluded that $m_{best}$ is related with the size of networks and system connectivity.

When nodes $v_{i}$ and $v_{j}$ are deployed at diagonal vertex of the square area, the farthest distance between a pair of nodes is obtained. The longest path proceeding from $v_{i}$ to $v_{j}$ is constant. Therefore, most hops from $v_{i}$ to $v_{j}$ in a random walk, which are approximately similar to $m_{best}$, are likely the longest path divided by radio range. With Algorithm 1, at $m_{best}$, our design considers the full connectivity of system, including the farthest situation in node deployment. Thus, the accumulated probability matrix may calculate all the node pairs’ walking probabilities, thereby maximizing the effectiveness of the new metric for relative distance. Consequently, the system may achieve the most desirable localization performance. This result may explain the reason why the lines in Figure 7 decline at the beginning. With the increase of *m* from one to $m_{best}$, this phenomenon fully utilizes system connectivity information stepwise. However, if *m* continues to increase, many repeat random walking may be considered into the accumulated probability matrix calculation. In such case, the system may cause an opposite effect in localization performance. The lines also present an upward trend.

Figure 7 also shows that lines go linearly when *m* becomes large, which confirms our conclusion that DD value may converge to the extreme value as *m* goes to infinity in Section 4.

#### 6.5.2. Effect of Anchor Density

**Figure 8:**
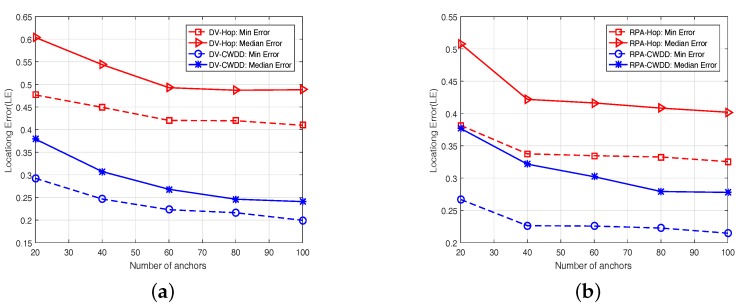
Different numbers of anchors: (**a**) Dv-based, (**b**) RPA-based.

In this simulation, we increase the anchor numbers from 20 to 100 in steps of 20 in the network, whereas other system configurations remain invariant. From Figure 8, we can see that minimum and median localization errors decline by the increase of anchor numbers for all methods. Therefore, a large number of anchors help improve system accuracy. From the Figure 8, we can see that CWDD-embedded methods show better performance than hop-based methods. The localization errors fall by an average of approximately 20% for DV-Hop and approximately 15% for RPA-Hop by applying CWDD. The maximum reduction of localization error is approximately 26% among all statistics.

#### 6.5.3. Effect of Node Density

**Figure 9:**
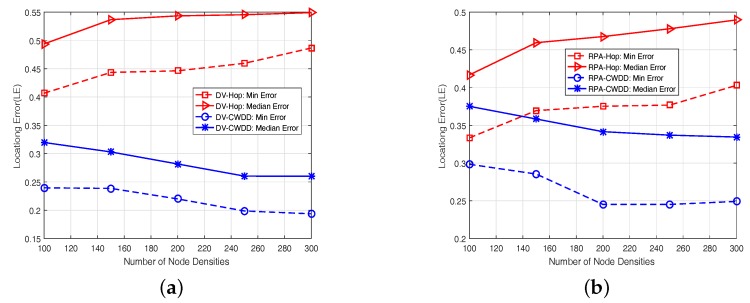
Different node densities: (**a**) Dv-based, (**b**) RPA-based.

In this experiment, we increase the number of nodes from 100 to 300 in steps of 50 without changing any configurations in a network map. Figure 9 shows the localization errors under different node densities for DV-based and RPA-based methods. CWDD-applied methods achieve approximately 22.7% and 15.8% performance gain from DV-Hop and RPA-Hop, respectively. It can be seen from Figure 9 that:

(1) For DV-Hop and RPA-Hop, the curve presents an increasing trend.That’s because the increased node density may cause the ambiguity problem in estimating coordinates, where nodes are mapped to the same positions.
(2) For CWDD-embedded methods, the accumulated probability matrix based on random-walk steps *m* can be applied to expand the discrimination between node pairs’ relative distance, where the shortest-path hops among node pairs are identical. More neighbors means richer network structure and higher connectivity, which can help to improve the accuracy of CWDD. Thus, the localization performance can be improved.

#### 6.5.4. Effect of System Scale

**Figure 10:**
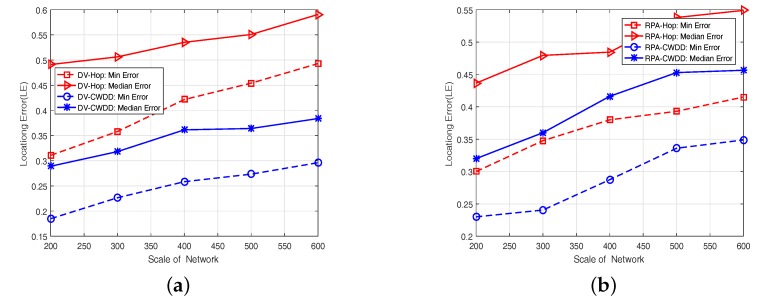
Different system scales: (**a**) Dv-based, (**b**) RPA-based.

In this experiment, we expand the dimension of the map from 200 m (in length and width) to 600 m in steps of 100 m. Note that the number of nodes in the network was increased proportionally to maintain the same node density. And we keep the number of anchors constant (20). Figure 10 shows that:

(1) CWDD-embedded approaches can achieve higher localization accuracy than that of original methods based on hops.
(2) With the enlargement of the system’s scale, all methods perform unfitting in localization accuracy because the number of anchors is not increased proportionally. Another reason is that the information of neighbor nodes is removed, due to the fixed communication range.

## 7. Conclusions

This paper mainly proposes a new metric for relative distance and a refined version, which are two range-free methods aiming at proving the node localization accuracy. Connectivity information is optimized, and our DD, which is a value of accumulated probabilities among node pairs in a random walk, is obtained as a new metric for relative distance. For further expansion, we provide a modified version called CWDD by adding proximity information. This new metric can be embedded into two classical hop-based localization algorithms for direct evaluation. Numerous simulations prove that CWDD methods improve system accuracy and provide a resolution for ambiguity problem based on several perspectives.

## Figures and Tables

**Table 1:** Default simulation configurations.

Parameter	Default Values Description
System Scale	700 m × 700 m
Noise Model	Logarithmic Attenuation Model
Number of Sensor Nodes	300, randomly deployed with uniform distribution
Proportion of Anchor Nodes	20%, randomly deployed
Radio Range	150 m

**Table 2:** Comparison: DD vs. CWDD.

	DD	CWDD
Localization Error (LE)	0.2118	0.186
Maximum Node Deviation	179.1175	144.8799
Minimum Node Deviation	1.1007	1.6635
Mean Node Deviation	27.5344	24.1856
